# Towards a community-driven definition of community wellbeing: A qualitative study of residents

**DOI:** 10.1371/journal.pone.0294721

**Published:** 2023-11-21

**Authors:** Camilla Michalski, Apira Ragunathan, Adrian Foster, Lief Pagalan, Casey Chu, Lori M. Diemert, John F. Helliwell, Diana Urajnik, Ruth Speidel, Tina Malti, Dianne Fierheller, Laura Fusca, Ian Zenlea, Scott McKean, Laura C. Rosella

**Affiliations:** 1 Population Health Analytics Lab, Dalla Lana School of Public Health, University of Toronto, Toronto, Ontario, Canada; 2 Schwartz Reisman Institute for Technology and Society, University of Toronto, Toronto, Ontario, Canada; 3 Institute for Better Health, Trillium Health Partners, Mississauga, Ontario, Canada; 4 Vancouver School of Economics, University of British Columbia, Vancouver, British Columbia, Canada; 5 Centre for Rural and Northern Health Research, Laurentian University, Sudbury, Ontario, Canada; 6 Laboratory for Social-Emotional Development and Intervention, Department of Psychology, University of Toronto, Toronto, Ontario, Canada; 7 Family and Child Health Initiative, Trillium Health Partners, Mississauga, Ontario, Canada; 8 Community Safety and Wellbeing Secretariat, Peel Region, Mississauga Ontario, Canada; 9 SafeTO, City of Toronto, Toronto, Ontario, Canada; 10 Laboratory Medicine and Pathobiology, Temerty Faculty of Medicine, University of Toronto, Toronto, Ontario, Canada; 11 ICES, Toronto, Ontario, Canada; St John’s University, UNITED STATES

## Abstract

**Background:**

Understanding what promotes or hinders a community’s capacity to serve the priorities of its residents is essential for the alignment of citizen needs and governance. Participatory approaches that engage community residents on the topic of community wellbeing are useful methods for defining outcomes that reflect a community’s goals and priorities. Using qualitative focus group methods, the aim of this study was to outline bottom-up definitions of community wellbeing from a diverse pool of community residents in Ontario, Canada.

**Methods:**

Semi-structured, two-hour group interviews were conducted with adult (≥18 years) participants (N = 15) residing in four communities across Canada’s largest province of Ontario. Participants were purposively selected from a pool of screening questionnaires to ensure diverse group compositions based on race, gender, age, and educational attainment. Interviews were thematically analysed using descriptive and interpretive methods to characterize resident conceptions of community wellbeing.

**Results:**

Focus group participants were between 18 and 75 years of age and most had lived in their local community for 5 or more years. Four major themes emerged: (1) a sense of community belonging is cultivated through shared spaces, routines, support, and identities; (2) a community constitutes the amenities and social contexts that enable residents to thrive; (3) effective regional decision-making must be community-informed; and (4) the wellbeing of a community relies on equal opportunities for engagement and participation.

**Conclusions:**

Residents described their communities and their associated wellbeing as a combination of accessible amenities and opportunities to engage without marginalization. This study underscores the value of participatory approaches in community wellbeing research, where the viewpoint and life experience of residents is used to inform local decision-making and service delivery. Future research will capture more diverse perspectives towards community belonging, particularly from community newcomers, for the development of regionally appropriate indicators of community wellbeing.

## Introduction

In recent years, there has been a growing recognition that the state of one’s community plays an important role in promoting wellbeing at both individual and societal levels [1]. What is termed “community wellbeing” refers to a framework of environmental, social, economic, political, cultural, and spiritual domains that shape a community’s goals and priorities [2–4]. Therefore, the aim of community wellbeing research is to understand these domains and evaluate the extent to which they advance or hinder a community’s capacity to fulfil the needs of its residents [3, 5]. Engaging in community wellbeing initiatives is a promising goal for local governments as it can enable the translation of community needs into tangible outcomes for governance by respecting citizens as experts on their own community [2]. Additionally, improving community wellbeing and belonging can serve to address important targets that have been identified by health and social decision-makers, including social isolation [6, 7].

Research on community wellbeing is largely centred on the development of indicators that aim to be objective tools of measurement [4, 8–10]. Such tools separate community wellbeing into discrete constructs (social, economic, political, cultural, etc.) that encompass the services, amenities, and social resources of the community (e.g., healthcare facilities, public transportation, or places of gathering) [5]. Such constructs are then used to create instruments that measure residents’ satisfaction, evaluation, and assessment of the constitutive components of wellbeing. While such tools do have the potential to bridge gaps between citizen needs and governance [10, 11], critics have argued that top-down instruments designed through subject matter expertise lack the contextual insight of the community (what Memon and Johnston refer to as “developed by scientists for scientists”) [12, 13]. More recently, researchers have underscored the importance of aligning community wellbeing tools (e.g., indicator measures and survey instruments) with the social and political values of the community in question to produce outcomes that are based on local evidence and reflect community perspectives [2, 12].

There is a growing consensus amongst community wellbeing researchers that frameworks and indicator systems are best defined in collaboration and co-design with community residents, actors, and organizations [2, 3, 12–14]. A recent contribution to community wellbeing as a theory is the incorporation of relational frameworks that recognize the interplay of community actors and their diverse priorities [3, 15]. Relational approaches place at their centre the relationships among individuals and groups within the community–antecedent to the singular individual [14, 16]. Relationality requires an understanding of how the sociodemographic and historical qualities of a community inform its goals, priorities, and norms [14, 16, 17]. Atkinson and colleagues discuss how the atomization of individuals as distinct units of measurement draws attention away from broader political structures that influence the state of communities and their potential to thrive [14]. Thereby, individual-level framing can result in key omissions concerning equity, sustainability, and culture when considering community wellbeing [14]. As such, the authors argue that individual data and statistical analysis may be limited in this respect, instead requiring participatory discussions that allow for the negotiation of interests in a democratic forum [14, 18].

This gap can be overcome by openly incorporating a plurality of perspectives directly from the community. These perspectives ensure that the outcomes produced will more meaningfully inform how societies operate, promoting more impactful progress [13]. Furthermore, participatory processes in indicator development can improve their relevance in policy and governance. Inductive approaches that generate domains directly from community actors have been demonstrated to (1) enable participatory engagement and transparency in regional decision-making, (2) promote the use of local evidence, (3) help define the shared goals and priorities of a community, and (4) help shift the focus towards practical outcomes for residents [2, 12]. Recent strides have been made in translating the results of such inductive approaches into policy implementation [19, 20].

In their work, researchers Kim, Kee, and Lee point to two drivers for the growing interest in community wellbeing as an instrument for policy: (1) there is a novel recognition that one-size-fits-all policies are insufficient in responding to community interests; and (2), especially in the face of limited public resources, national governments may be more inclined to hand off community wellbeing pursuits to local governments [21]. Such interest has created a unique opportunity to engage community actors across a variety of regional contexts and develop relative priorities. However, while numerous tools measure community wellbeing [4, 8–10, 22, 23], there remains a paucity of research that engages directly with community residents to define key areas of measurement.

Using semi-structured focus group interviews and thematic analysis methods, our aim for this study was to outline key themes and perspectives towards community wellbeing from a diverse pool of residents in four communities in Canada’s largest province of Ontario. Our goals were to (1) inform a framework of community wellbeing relevant to Ontario communities and (2) produce bottom-up, community-derived themes of community wellbeing using participatory and inductive approaches. While we present here our study of community wellbeing themes, in future work we will use the themes to develop and implement an organized framework of community wellbeing that can be scaled across Canada.

## Materials and methods

### Setting

To capture perspectives across diverse regional contexts, we recruited participants in four Ontario municipalities that vary across geography, population density, diversity, public infrastructure, and development: (1) The City of Toronto, (2) The Regional Municipality of Peel, (3) The City of Greater Sudbury, and (4) The City of Thunder Bay.

The City of Toronto is Ontario’s largest metropolitan centre, with high population and resource density, cultural diversity, and transit infrastructure [24]. The Regional Municipality of Peel is a mixed urban and suburban community, has a culturally diverse population, and is one of Ontario’s fastest growing regional municipalities. By comparison, Greater Sudbury and Thunder Bay support lower density populations and are geographically distant from federal and provincial decision-making centres (i.e., Ottawa and Toronto). The 2021 Census reported that while the majority populations of Greater Sudbury and Thunder Bay are White, these communities also have Indigenous (First Nations, Métis, and Inuit) populations greater than the provincial average [24].

### Participant recruitment

Between May and July 2022, potential participants were invited to complete a focus group screening questionnaire to confirm their eligibility (i.e., ≥18 years of age and living in one of the four regions), capture key demographic information, and ask about necessary interview accommodations (e.g., live transcription, technical support). The screening questionnaire was distributed using social media advertising and to participants of The Community Wellbeing Survey; a cross-sectional, online survey administered by the research team within our four focal communities in Fall 2021. We leveraged several networks to expand recruitment: (1) community organizations and advisory tables connected to the research team, (2) community organizations identified via online searches, and (3) email listservs and social media advertising. Recruitment materials were provided in English.

Interview participants were selected from screening questionnaire respondents. To prioritize diverse perspectives, we selected interview participants through stratified sampling by region and demographic characteristics. The focus group composition was checked so at least 50% were women, were racialized (i.e., Black, Indigenous, People of Colour), and there was a range in age and educational attainment.

### Interview guide development

Focus groups were two hours in duration and held virtually via videocall in English with one facilitator and two co-facilitators (CM (f); AR (f); LP (m); CC (f)). Four focus group interviews, one for each region, were conducted with 3–4 participants each (N = 15). The aim of the focus group interview guide was to obtain perceptions of community wellbeing from participants, considering their personal experiences and their perceptions of the experiences of others in their community. The semi-structured guide flowed through four general community wellbeing domains: social, physical, environmental, and political (S1 Table). Questions, prompts, and domains were adapted from validated surveys and questionnaires, as identified in a literature review of community wellbeing [4, 8, 15, 25, 26], and further refined based on responses from The Community Wellbeing Survey. The interview guide focused on the geographically bound concept of local community (e.g., the places where one lives, socializes, and accesses resources).

To facilitate idea generation and discussion, a collage was shared with participants on screen depicting different community-related concepts such as informal social support (e.g., neighbours shovelling snow together), walkability/transportation (e.g., bike lanes, wheelchair ramps, public transport), and necessary amenities (e.g., grocery stores, health clinics) (S1 Fig). Participants were then prompted to use the collage as a jumping off point to consider ideas pertaining to community wellbeing (“what is missing from the collage?”, “does this resonate with your community?”, etc.).

To orient participants to consider how social and physical components relate to the wellbeing of their communities, each section started with a brainstorming activity in which a prompt was shown on screen and participants were instructed to write down all the words that came to mind. After 30 seconds, participants shared their lists. Participants then answered questions regarding their individual and intersubjective experiences (i.e., shared perceptions, feelings, or meanings) within the social and physical components named in the lists.

Interviews were audio recorded and transcribed, and respondents were compensated with a Can$50 gift card.

### Qualitative analysis

Thematic analysis was conducted using NVivo 12 (QSR International™). Code generating methods started with provisional coding, in which a preliminary codebook was developed *a priori* to abstract and synthesize the data based on a theoretical framework informed by existing community wellbeing literature (e.g., using keywords such as “affordability”, “distance”, “basic needs”, “membership”, “recognition”, “trust”, “safety”, “opportunities to socialize”, “tensions”, etc.). Following an initial review of the transcripts, further provisional codes were added. At this stage, more detailed descriptions of each code (i.e., defining inclusion and exclusion criteria) were also specified. Using these provisional codes, interview transcripts were coded again and thematically analysed simultaneously by two coders using an amalgamation of descriptive and interpretive methods [27, 28]. Agreement on coding patterns was sought through intensive group discussion with two additional interview co-facilitators to find more salient coding categories for second cycle coding. Transcripts were then re-coded, followed by a second group discussion to finalize themes. Using NVivo 12, finalized codes were compiled into a data display, where quotations were organized across coding patterns that reflected key themes shared within the focus group interviews. The data display served as the central repository of qualitative data to draw conclusions.

### Research ethics statement

The University of Toronto’s Human Research Ethics Board (REB) approved the research protocol (Protocol Reference #41692). All participants were required to provide informed written and verbal consent before initiating the interview. Participants were asked to read through and provide written consent to the informed consent protocol, outlining how their data would be stored and how they were free to opt out of any question or have their responses removed. Participants were reminded that their participation was voluntary and that all of their responses would remain confidential and anonymized. Verbal consent was re-affirmed and witnessed by the interview co-facilitators (CM; AR; LP; CC).

## Results

### Study sample

We conducted four regional focus groups with residents from Greater Sudbury, Peel, Thunder Bay, and Toronto, with each focus group composed of three to four participants (N = 15). Participants ranged from 18 to 75 years of age, with most respondents being between the ages of 26 and 40. Most participants were women. Black, East Asian, Southeast Asian, Indigenous, and South Asian participants were included in the focus groups, but the majority were White. The plurality of respondents had an undergraduate degree, and almost all participants had lived in their local communities for five or more years.

### Thematic analysis

Using a qualitative and interpretative approach, community wellbeing was conceptualized into four broad themes based on the insights of the participants (Table 1). The four themes presented in this section are: (1) a sense of community belonging is cultivated through shared spaces, routines, support, and identities; (2) a community constitutes the amenities and social contexts that enable residents to thrive; (3) effective regional decision-making must be community-informed; and (4) the wellbeing of a community relies on equal opportunity for engagement and participation. Each theme is divided into sub-themes to clarify the diverse views within each theme, and each sub-theme is presented with direct quotations from interviews as qualitative evidence.

**Table 1:** Summary of main community wellbeing themes and sub-themes, with quotations as qualitative evidence.

	Main Themes	Sub-themes	Quotations
**1**	**A sense of community belonging is cultivated through shared spaces, routines, support, and identities**	1.1 Familiarity with spaces	*For me*, *[it’s] about consistency*, *of being really accessible… Having the space to do that has always been in my head*.
		1.2 Recognition	*I feel as though I belong when I’m noticed*. *But in a good way*.
		1.3 Helping and engaging with neighbours	*I think having deeper relationships with neighbours and people in your community gives you a sense of belonging*.
		1.4 Shared identity and exposure to different identities	*A lot of people I connect with*, *they’re either through work or school*.* *.* *. *Everyone’s around your age*, *so it’s easy to meet people*. *It’s easy to really sympathize and understand people there*.
**2**	**A community constitutes the amenities and social contexts that enable residents to thrive**	2.1 Important amenities	*We’ve pretty much got everything everyone needs here*, *really*.* *.* *. *We could survive just in our own little community*.
		2.2 Access to amenities	*There are a lot of services that are overwhelmed that cannot take any more people*, *and they’re very limited in their funding*. *Indigenous peoples or newcomers*, *even if they could access the service*, *probably would be sitting on a waiting list forever*.
**3**	**Effective regional decision-making must be community-informed**		*Talk with your community*. *Get permission*. *It’s not going to kill you to get permission from the Indigenous communities*. *And*, *you know*, *just work with everybody*.
**4**	**The wellbeing of a community relies on equal opportunity for engagement and participation**		*We understand that in the society in which we continue to operate*, *networking [and] social connections [are] important not only for wellbeing but for other benefits [as well]*. *So*, *I see it as a privilege*.
			*Right now [in one particular community–redacted detail]*, *there’s somebody on every corner asking for money*, *right… I should be figuring out how to make everybody have what I have*.

#### Theme 1: A sense of community belonging is cultivated through shared spaces, routines, support, and identities

When respondents were asked what makes them feel like they do or do not belong to their local community, the most common responses were related to a sense of familiarity with spaces and people. Familiarity with people was described as shared routines (e.g., run-ins on the street, waiting for the same public transport, etc.), support (e.g., sharing community news, offering to help with property maintenance, etc.), and shared identity (e.g., cultural group, age group, etc.).

*1*.*1 Familiarity with spaces*. Having routine and safe access to spaces in the community was described as cultivating a sense of connection to one’s physical and geographical place, *“for me*, *[it’s] about consistency*, *of being really accessible… and having the space to do that has always been in my head*. *A fundamental aspect—like I’ve got to find spaces”*.

*1*.*2 Recognition*: *“I feel like I belong when I’m noticed”*. In line with routinely accessing spaces, frequently visiting the same spaces was connected to developing recognition, familiarity, and rapport with people simultaneously visiting those same spaces:

*You can be a regular somewhere… [At] restaurants and stuff like that. And then you start making friends, and you expand your social circle, which I feel is really important… Like you see familiar faces*.

In other words, noticing familiar faces, and in turn feeling noticed, fostered a sense of belonging:

*I feel as though I belong when I’m noticed. But in a good way. Because I’m a commuter, there will be regulars on my routes. But the day they engage with you is the day where you realize that they’re looking out for you. I was late once. I was not on the route. The next day, “Oh… are you okay? Because I didn’t see you on your way to work yesterday.”*

*1*.*3 Helping and engaging with neighbours*. Neighbourly support was described as an antidote to isolation. For some participants, a sense of community belonging required more than just the presence of others, but to actively help one another. Having a common purpose was viewed as a motivating driver to support one another, which in turn fostered a sense of belonging:

*Having deeper relationships with people in your community gives you a sense of belonging… You know, you could reach out if they have the same situation as you. So, I feel like that could make you feel that you have people to rely on in your community rather than feeling isolated and alone*.

*1*.*4 Shared identity and exposure to different identities*. Participants viewed shared identity and group membership as strong determinants for community belonging. The most shared identities (in terms of being a catalyst of community formation) were specific life stages and social roles (i.e., student, parent), age groups, ethnicities, and shared political views:

*A lot of people I connect with, they’re either through work or school… Everyone’s around your age, so it’s easy to meet people. It’s easy to really sympathize and understand people there*.

While membership to specific groups was consistently described as central to fostering a sense of belonging, participants also noted how defining those groups too narrowly could create siloed communities. One participant reflected on events in their community and noted:

*All these events are tied so intimately with culture. Yes, you do have members of the community organising events like this. But again, there’s still some exclusivity because of how they communicate the events, how they promote it, how they recruit, and where you can get information*.

Relating to siloed communities, many participants expressed feeling that there was a lack of opportunity to connect with community members who they do not share an identity with. Specifically, participants pointed to a lack of broader community events and event spaces. For instance, one participant mentioned wanting to interact with people from other backgrounds:

Where are the people of different cultures, and why are we not seeing each other? … It’s not like we’re integrated [or that] we see each other a lot. So, I would like to have more opportunities to have more diverse relationships.

Moreover, there were sentiments of a deteriorating sense of collectivism at a larger societal level:

*I think we try to mind our own business more than we try to help out anymore. I think that’s just in a general sense. I wouldn’t say that that’s specific to my community. I think it’s just generally*.

#### Theme 2: A community constitutes the amenities and social contexts that enable residents to thrive

*2*.*1 The availability of important amenities*. Participants identified several amenities that they considered key components of their local communities, and many defined the geographical bounds of their local communities through the presence of necessary amenities. Local communities were commonly defined as the space in which one could meet the core needs of daily living, *“We’ve pretty much got everything everyone needs here*, *really*… *We could survive just in our own little community”*.

Examples of amenities that were identified as key were grocery stores, places of worship, health care centres, recreation centres, and public greenspace. For instance, when identifying spaces and places that contributed to the wellbeing of their communities, a commonly expressed sentiment was how access to greenspace contributed to the physical, mental, and spiritual health of community residents, *“I really enjoy the trails*, *distinct from the park*, *because it just allows you to disconnect and unwind for a bit”*.

*2*.*2 Access to amenities*. The accessibility of amenities was described across four axes: availability, affordability, physical access, and proximity.

The availability (and unavailability) of health centres was a central discussion point across all groups, and notably, the affordability of mental health care. Affordability was also discussed in relation to recreational activities (e.g., not being able to afford fees to attend classes) and housing (i.e., that this should a met need for all community members). One participant noted the unique barriers to access for Indigenous and recently settled community members:

*There are a lot of services that are overwhelmed that cannot take any more people, and they’re very limited in their funding. Indigenous peoples or newcomers, even if they could access the service, probably would be sitting on a waiting list forever*.

Physical access was discussed in terms of maintenance (e.g., maintaining clear sidewalks during winter), and a shared responsibility to contribute to the maintenance of community spaces. Maintenance of one’s community was viewed as having intrinsic value–demonstrating a level of care and respect for where and with whom one lives:

*Everybody’s on the same mission to [keep] the place clean and safe for everybody to enjoy… Having people that care about the same kind of values … that you care about for your community is important*.

Additionally, the importance of functional transportation was seen as a necessary public good to guarantee residents’ proximal access. However, sentiments differed across geographies. Among residents that lived outside high-density population centres, public transportation was viewed as a necessary service to connect physically distant communities. On the other hand, views from residents living in high-density communities noted traffic and congestion which hindered mobility within their community.

#### Theme 3: Effective community decision-making must be community-informed

Residents felt their needs and wants were not reflected in regional decision-making. Specifically, many residents said that their community leadership ignored pressing gaps in basic needs, namely unaffordable housing, long wait times for health care, and unaffordable mental health services, *“The city would rather spend money on pretty things to make [the city] look good than actually fix the potholes where people can get hurt”*.

One resident stated:

*The politicians need to get this little catchphrase out of their vocabulary altogether, “It’s not on my agenda.” Just because it’s not on your agenda doesn’t mean it’s not on everyone else’s. To me, affordable housing is really lacking… [as is] food security*.

A common thread was a desire to increase communications between community residents and decision-makers. For example, when asked what ought to be prioritized in local decision-making, one resident stated:

*Definitely the preservation of the lands, and truly respecting the Indigenous land by not just bulldozing it to build new homes. Like do some actual planning. Talk with your community. Get permission. It’s not going to kill you to get permission from the Indigenous communities. And, you know, just work with everybody*.

Another participant noted that even when residents’ opinions are collected, the decisions that follow are not necessarily informed by these consultations, *“I’m laughing because city council will hire a consultant*, *and then the consultant will give a report*, *and then they’ll do whatever they want anyway”*.

Furthermore, many participants felt the most privileged tended to persuade decisions:

*So poor people, their concern is, how do I get food on the table? Middle class people say, is it nutritious? The wealthy say, is it pretty? So, the decision-makers say, is it pretty? … [Decision-makers need to understand] what happens when we don’t have a liveable wage*.

One participant from a focus group in Northern Ontario discussed how provincial policies did little to bridge remote Northern communities with decision-making centres largely located in the South:

*The ministry is based in Toronto [and] very frequently forgets that we exist. The premier thinks that we’re North Bay and not Thunder Bay… A lot of issues, we would say, fall upon the provincial government and not the local government… It results in nobody doing anything a lot of the time*.

#### Theme 4: The wellbeing of a community relies on equal opportunity for engagement and participation: “Flourishing should not be a privilege”

As established, community wellbeing was perceived in relation to belonging, participation, spaces, access, and leadership. However, to flourish within one’s community, one must have sufficient opportunities to participate. One participant highlighted this connection, noting how community involvement is a privilege not afforded to all:

*There is an advantage to being well-connected in the community… We understand that in the society in which we continue to operate, networking [and] social connections [are] important not only for wellbeing but for other benefits [as well]. So, I see it as a privilege. You want to be well-connected, but you can’t. So, if you’re not as well-connected because you continue to work, you might lose out on other opportunities. Because to be well-connected is also to be well-informed. And information is a resource, and information is not always the easiest to come by either, especially when it’s the type of information that impacts your livelihood and your community, which in turn impacts your wellbeing*.

This same participant highlighted the difference in events hosted in the more privileged community they work in, versus the events hosted in the less privileged community they live in:

*But the priorities of [the more privileged] community are so different from the ones [in my less privileged community]… They can spend more time looking after their health… Whereas in [my community], you have more events geared toward language learning or … learning how to navigate the city… What we want to work towards is not the same for everyone*.

Most participants shared an intersubjective perspective wherein they recognized an inequitable distribution of resources within their communities. One participant remarked:

*I know there’s some places in our local community that it’s hard. Like right now [in one particular community–redacted detail], there’s somebody on every corner asking for money, right… I should be figuring out how to make everybody have what I have*.

Another participant discussed how historical and contemporary discrimination created disparities in how community members could flourish, particularly for Indigenous residents in their community:

*Acknowledging that the impacts of residential schools [and] the Sixties Scoop has done a lot of intergenerational trauma to a very large portion of Thunder Bay… A number of institutions in Thunder Bay still have systemically racist policies [and] practices*.

Notably, one resident felt the relative scarcity of resources created a tension between prioritising the needs of local communities and aiding others:

*Take care of the people that live in your own community first before you start looking at the world. I agree… I’m not saying that we can’t help people in the world, but at least help the ones at home as well*.

## Discussion

Our qualitative study identified components of community wellbeing through resident focus groups in four Ontario communities (Greater Sudbury, Peel, Thunder Bay, and Toronto). We identified four themes of community wellbeing: (1) a sense of community belonging is cultivated through shared spaces, routines, support, and identities; (2) a community constitutes the amenities and social contexts that enable residents to thrive; (3) effective regional decision-making must be community-informed; and (4) the wellbeing of a community relies on equal opportunities for engagement and participation. Each theme encompassed perspectives on shared community amenities, values, and structures that residents saw as essential when considering the wellbeing of their community.

The relationship between wellbeing and having access to diverse and rich social networks is well-established [29–31]. Maintaining strong social connections is a meaningful social determinant of health, providing useful insulation against loneliness, which has been associated with premature mortality and worsened mental wellbeing [6, 7]. Participants viewed social belonging, recognition, and support as key facilitators for community wellbeing (Theme 1). Social communities were described as physical spaces where one could develop strong bonds and facilitate new relationships. These places included public transit, schools, places of work, community centres, and community events such as festivals or cultural activities. Importantly, participants defined social communities as systems of mutual support, where acts of aid would be reciprocated in turn. Therefore, there was a responsibility on behalf of residents to actively engage with their community (e.g., volunteer, support neighbours when asked) and facilitate community wellbeing.

However, some residents questioned the sustainability and resilience of their social communities. For instance, there was a view amongst some respondents that the strength of community belonging was weakening over time–citing a loss of collective identity. While we cannot interpret whether these sentiments signify a perceived or actual change in the strength of collective identity, it demonstrates that there is a need for community arrangements that facilitate social connection and shared purpose [7]. Recent research on social portfolios indicates that creating and cultivating shared community spaces can mitigate feelings of social isolation by enabling community residents to maintain diverse and regular social communities [29]. In our focus groups, an example raised was to make places of gathering or community-oriented activities more accessible for residents by removing barriers that exclude specific groups.

In addition to the social aspects of a community, community wellbeing is also dependent on the creation and maintenance of physical facilities that allow residents to fulfil their daily needs (e.g., food, clothing, shelter, and healthcare) [4]. Services and amenities were identified as key contributors to community wellbeing, with some participants defining the boundaries of their community through the physical amenities that they frequented every day (Theme 2). Participants identified barriers of availability, affordability, physical access, and proximity that contributed to inequities in wellbeing. Services and amenities were often described as being “overwhelmed” or lacking the capacity to provide service to all members of the community. As has been demonstrated, the disconnect between residents’ needs and the ability to satisfy them inhibits “place attachment” and affective ties to one’s community [32]. Projects of community development that engage with residents’ needs, as well as their social and cultural interaction with physical space, have been shown to bolster a “sense of place” in communities [33].

The capacity to lend one’s perspective and be a meaningful contributor to local governance and decision-making were clearly defined components of community wellbeing in our focus groups (Theme 3). Participants identified the necessity of democratic processes that allowed for the alignment of their priorities and their leadership’s capacity to act on them. However, residents expressed that they were rarely consulted in regional decision-making. A commonly shared view was that local leadership did little to respond to gaps in basic needs, stating that decision-making often reflected the interests of those who had the time, resources, and connections to persuade said decisions.

Considerations must therefore be made to include a plurality of diverse voices in discussions of community wellbeing. It has been routinely demonstrated that the opportunity to provide one’s opinion and contribute to community decision-making is hindered by those same inequities that must be deliberated–such as systemic racism, marginalization, and exclusion [34, 35]. This is significant as disengagement with local leadership and decision-making have been highlighted as key deterrents to community wellbeing [8, 36, 37]. Principles of co-design, where knowledge is shared and integrated among residents and stakeholders, could serve as a model framework for community wellbeing planning so that decision-making is inclusive and participatory [38]. In Canada for instance, initiatives such as British Columbia’s Healthy Community Strategy have begun to incorporate community wellbeing principles to inform equitable and sustainable policy in the province [19]. In a similar vein, Ontarian provincial legislation has mandated all municipalities to develop evidence-informed Community Safety and Wellbeing Plans [20]. With governments and municipalities increasingly developing community wellbeing plans, our findings point towards both a need for, and the benefits of, including community residents in such efforts, especially considering historical and systemic exclusion.

Our fourth theme (subtitled “Flourishing should not be a privilege”), rather than being a distinct component of community wellbeing, revealed itself to be a cross-cutting layer that informed our other themes. The opportunity to engage in one’s community, whether it be socially, politically, or logistically, was seen as a privilege not afforded to all community residents. This privilege was resource-dependent, with those lacking time, assets, or social capital being excluded or otherwise preoccupied with addressing their daily needs.

Participants discussed the systemic and historical nature of this privilege, with discriminatory and exclusionary practices being key determinants. For instance, participants shared how communities who have endured economic disadvantage, social marginalization, and racism (e.g., Indigenous, recently settled, and racialized communities) experience inequities in access to resources, participation in community-decision making, and the privilege to flourish in their communities. Regionality and geographical context were also top of mind, with participants from Northern Ontario (Greater Sudbury and Thunder Bay) regularly describing a disconnect between their communities’ needs and the priorities of provincial and federal decision-makers in more distant cities.

The inequities identified in our focus groups reflect observations that communities exist as complex systems, where collective and individual interests often exist in a state of conflict [14, 17, 35, 39]. As has been observed across community wellbeing research, such conflicts often reflect the structural and historical inequities that exist within the community [35]. Relational approaches that engage with diverse and historically-informed perspectives are essential in producing equitable and sustainable outcomes [14]. Atkinson and colleagues have argued that opening discursive spaces that allow for the exploration of said conflicts serves to produce practical outcomes for residents as well as enable creation of community wellbeing itself [14, 18]. In their article, Trickett and colleagues assert that effective community interventions, including community wellbeing, necessitate a transition from individual- to community-level outcomes [17]. Individual-level measures in local planning, while having value at the micro-level, are often insufficient in addressing macro-level problems that emerge from the complexity of social relationships, especially considering social inequities [14, 16, 17, 39]. That is not to say that relational approaches ignore individual needs, but rather that they recognize that novel properties emerge when individuals are organized into collectives based on shared place, values, and histories [14, 39].

Researchers have begun to question the “scientific objectivity” of indicator systems [12, 13]. Indeed, how researchers choose which instruments to use is often constrained by interests related to research and may not reflect the realities faced by the communities in question [13]. The range of potential regional, social, and historical contexts therefore requires measures of community wellbeing to produce outcomes that are based on local evidence and reflect community perspectives [12, 13]. We agree that participatory indicator development, where communities are consulted in the domain-selection process, can help align research outcomes and the priorities of community actors [12]. Without a rigorous and systematic approach to understanding community wellbeing from a broad range of stakeholders, there is a risk that efforts aimed at enhancing community wellbeing will not respond to the genuine needs of the community or adapt equally across different subpopulations.

### Strengths and limitations

This research was strengthened by its use of well-established qualitative methods, within both our focus group interviews and thematic analysis. Principles of *trustworthiness* were integrated throughout [28]. Specifically, investigator triangulation was conducted with two coders to help minimize bias, then discussed more generally with two analysts who facilitated the interviews. All focus groups were conducted with a lead facilitator and two co-facilitators, which allowed for reflexive notetaking throughout data collection, analysis, and interpretation. Direct consultation with community residents permitted multiple stakeholders’ and subpopulations’ needs to be understood, demonstrating a path for subsequent opportunities to improve their communities.

Despite its strengths, this study has limitations in its design and analysis. Member checking of the data display was not performed, and the generalizability of the results may be limited by a relatively small sample. While member checking was not possible, the data display was discussed with local stakeholders and organizations to ensure the findings resonated with local communities. As mentioned, in an attempt to ensure that participants had a breadth of community experiences (both before and during the pandemic) to draw from during the interviews, we attempted to capture a diverse sample across age, ethnicity, gender, and time lived in one’s local community. However, the heterogeneity within and across groups was limited by the small sample and convenience sampling approach (e.g., because participants were recruited online, community residents with limited technological access were excluded). Indeed, the majority of participants were ultimately between the ages of 26 and 40, women, and White. As such, there may be differences when generalizing to other communities across Canada. Future work is planned to expand on the methods and recruitment outlined in the paper to emphasize additional perspectives toward community belonging (i.e., newcomer, recently settled, and migrant perspectives) and prioritize more inclusive modes of recruitment (e.g., postering, community outreach, etc.).

## Conclusion

Using qualitative methods, this study articulated themes of community wellbeing as they were experienced by a diverse group of residents in Ontario, Canada. We identified four themes of community wellbeing which spanned topics of social connection, physical facilities, inclusive decision-making, and equity. In describing each of these themes, participants also highlighted key barriers and inequities. Indeed, the opportunity to flourish in one’s community was shaped by local contexts and material stability, resulting in feelings of marginalization. However, participants still expressed an interest and desire towards engaging further in efforts that pertain to their community, indicating the importance of including mechanisms to integrate community voices and enhance collaboration between decision-makers and residents.

In recent years, there has been a growing motivation to move past economic measures of progress, and towards contextual indicators of community wellbeing. This study underscores the value of participatory and inclusive approaches in community wellbeing research, where the viewpoint and life experience of residents is used to shape local decision-making and service delivery. Our findings point towards a need for including community residents in such efforts, especially considering historical and systemic exclusion. As local governments gain interest in understanding the wellbeing of their communities, such efforts should recognize community residents as experts on their own needs and value their essential role in building communities that support better lives.

## Supporting information

S1 Table Focus group interview guide. (DOCX)

S1 Fig Focus group collage. (TIF)
