# Supplementary material for: Towards a community-driven definition of community wellbeing: A qualitative study of residents
Source: PLoS One. 2023 Nov 21;18(11):e0294721. doi: 10.1371/journal.pone.0294721 (PMC10662708; doi:10.1371/journal.pone.0294721)
Supplement: S1 Table — (DOCX) [file pone.0294721.s001.docx]

**S1 Table.** Focus group interview guide.

| **Questions for Residents** |
| --- |
| **Part 1: Introduction** |
| We’re going to be talking about community wellbeing, which refers to the *overall* wellbeing of the people in a community & includes how different parts of the community work together. Today we’ll be asking you to share your experiences within your own local communities.  ● **[Introductions]** To begin, let’s go around the table & introduce ourselves. State your first name, and how long you’ve been living in your current community. I’ll go by the order on my screen, starting with the research team. |
| **Part 2: Warm-up Brainstorm** |
| **[Brainstorm – General prompt]** Thank you everyone. We’re now going to do a quick brainstorming activity together. Throughout the two hours we’ll do a total of three brainstorms - this first one is general, and the two that come later will relate to specific aspects of the local community.  For each of the brainstorming activities, we’ll show a prompt up on the screen and give everyone 30 seconds to jot down the first words that come to mind in the Zoom chat box. You can access the chat box by clicking the chat icon at the bottom bar of the screen. After 30 seconds, we’ll ask everyone to press enter at the same time and start a short discussion.  ● Starting with our first brainstorm, what words come to mind when you hear the term ‘local community’?  ● That’s 30 seconds - let’s stop writing and start sharing. Could everyone please press enter? Let’s start with *[participant X].*  *[Primary: Give every participant up to 1 minute to make a remark]*  ● **[Brainstorm – Collage]** Looking at this collage (see Appendix 2), do you see anything you want to expand on?  **[Local community definition]** *Thanks for sharing. To wrap up this first brainstorm, our research team has also landed on a similar definition, which is that the local community is the place where you live, play, and get most of your necessary amenities from. Sometimes, but not always, our local communities also include where we work.* |
| **Part 3: Social Domain** |
| **[Brainstorm – Social domain prompt]** So that was our general brainstorm and now we’ll focus in on specific aspects of community, starting with the social aspect. We’ll use the same activity structure – please jot your thoughts in the chat box, but don’t press enter until we ask you to at the end.  ● Who are the people in your local community that make it feel (or not feel) like a community?  ● What do people do that makes this local area feel (or not feel) like a community?  *Probe: To clarify, this can include people that you see often, that you’re close with, or people that you have brief interactions with.*  ● Alright, let’s stop writing and start sharing. Please press send. Let’s start with ______.  **Social examples** (only if needed): Emotional support, tangible resource support (e.g., lending tools, providing food, childcare, school/extracurricular transport), short interactions (e.g., stopping to chat, familiar barista), organized events (e.g., block parties, garage sales), safety (e.g., neighbourhood watch), disaster/pandemic-driven, snow shovelling, intergenerational activities, education |
| ● **[Social – Belonging]** Could you share some examples of what makes you feel like you belong, or what makes you feel isolated?  *Probe: Remember, you can think about and share very casual interactions, as well as closer/more supportive interactions.*  ● **[Social – Priorities]** What types of interactions do you value most or wish you had more of? |
| ● **[Social – Access]** Is it equally easy (or equally hard) for everyone in the local community to find and maintain social connections? In what ways? Please share examples. |
| **Part 4: Physical Domain** |
| **[Brainstorm – Physical domain prompt]** Great. Now we’ll move on to our third and final brainstorm, which focuses on another aspect of community living – the physical environment. Again, we’ll give everyone 30 seconds.  ● What physical structures, spaces, and services are related to your community’s wellbeing?  ● Think of where you like to go, where you need to go, where you go often, or where people come together in your local community.  *Probe: Remember, these can be public spaces and private spaces. As for services, you can think about services you use daily, monthly, or only when we need it.*  ● Alright, please press send. Let’s start with ______.  **Physical examples** (only if needed): Greenspace, transport (road conditions, parking, traffic, public transport access/quality), walkability (lighting, proximity of amenities, sidewalks), cultural/spiritual spaces, recreation/leisure/ entertainment spaces (community centres, parks, sport centres), spaces offering necessary amenities (grocery stores, schools/childcare centres, banks, health centres, cost of living/affordability) |
| ● **[Physical – Priorities]** Thinking about physical structures, spaces, and services, which are the most important to you and why? |
| ● **[Physical – Access]** Do you have more positive or more negative experiences compared to other people? Please provide examples.  *Probe: What is it that makes people feel more or less comfortable using spaces?* |
| **Part 5: Political Domain** |
| ● **[Brainstorm – Political]** Finally, we’d like to talk about improving local communities. What changes have been made in your local community, or where is there room for improvement? These examples can be big or small.  *Probe 1: Note that this doesn’t have to be something that you are affected by or involved in. It can also be an issue or change that impacted someone else.*  *Probe 2 (optional): You can think of what things contribute to the wellbeing of your local community, or things that damage it.*  **Change examples** (only if needed): Pandemic/ disaster-related (e.g., organized mutual aid support), PTA, fundraisers, bylaw implementation (e.g., stop sign addition) |
| ● **[Political – Influence]** Who has, or who do you think could, create overall positive change in your community?  *Probe (optional): These people could be other residents, people that work/volunteer for local community organization, or municipal leaders.*  ● Why do you think these people are influential? |
| ● **[Political – Access]** When people want different things to change, is everyone’s voice equal? Whose voices are heard the most? |
| **Part 6: Conclusion** |
| That brings us to the last section. We want to share people’s opinions and voices with policymakers.  ● **[Indicators]** If you could share one or two pieces of information about your local community, what information would you like policymakers to have?  *Probe 1 (optional): For example, there may be things that policymakers don’t always know about your community because they don’t live there. What would you like to share with them?\*  *Probe 2 (optional): You can think of improvements that need to be made.* |
| ● Wrapping up, does anyone have any final thoughts that have not yet been brought up? |
