# Supplementary figures and images for: Towards a community-driven definition of community wellbeing: A qualitative study of residents
Source: PLoS One. 2023 Nov 21;18(11):e0294721. doi: 10.1371/journal.pone.0294721 (PMC10662708; doi:10.1371/journal.pone.0294721)

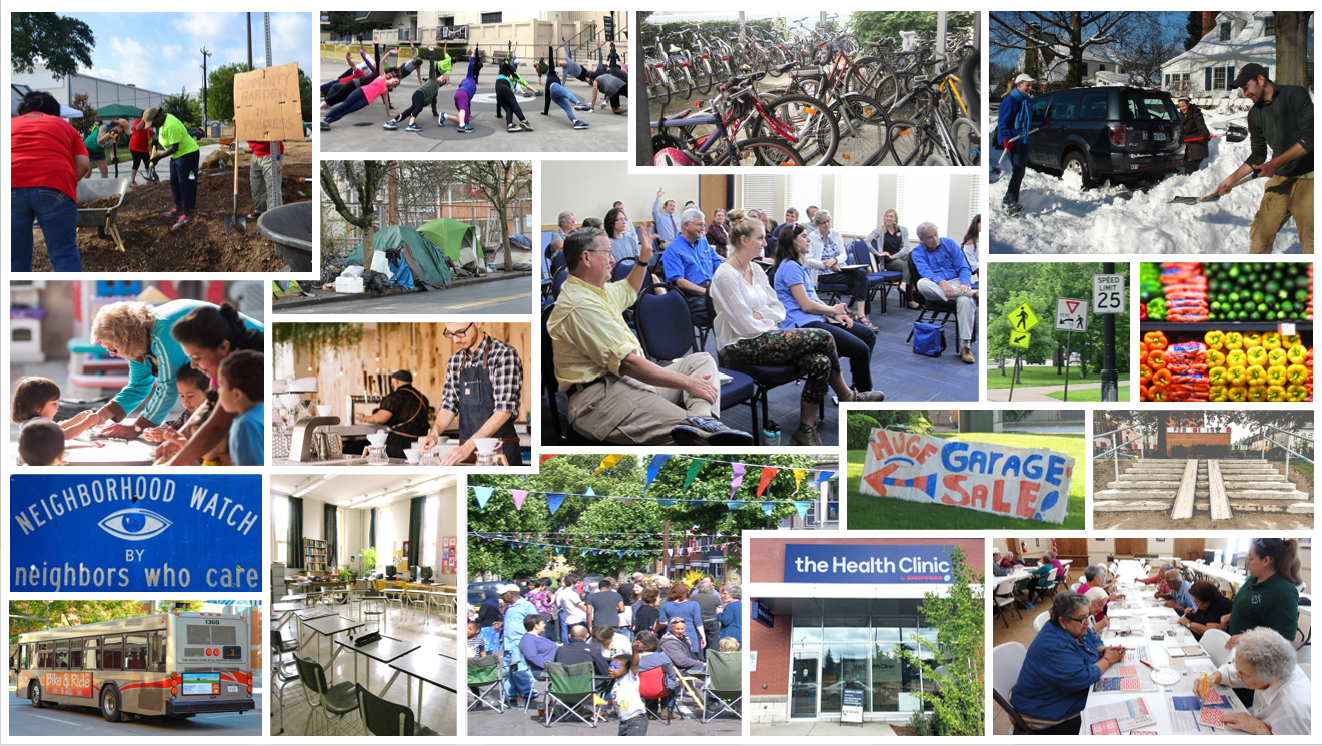

Supplement: S1 Fig — (TIF) [file pone.0294721.s002.tif]
